# Clinical and Laboratory Findings by Serum Vitamin D Levels in Children with COVID-19

**DOI:** 10.5152/eurasianjmed.2022.22213

**Published:** 2022-10-01

**Authors:** Ayse Ozden, Hakan Doneray, Emine Hafize Erdeniz, Konca Altinkaynak, Hakan Igan

**Affiliations:** 1 Department of Pediatric Endocrinology, Erzurum Regional Training & Research Hospital, Erzurum, Turkey; 2 Department of Pediatric Endocrinology, Atatürk University Faculty of Medicine, Erzurum, Turkey; 3 Clinical Research Development and Design Application and Research Center, Atatürk University, Erzurum, Turkey; 4 Department of Pediatric Infectious Diseases, Erzurum Regional Training & Research Hospital, Erzurum, Turkey; 5 Department of Biochemistry, Erzurum Regional Training & Research Hospital, Erzurum, Turkey; 6 Department of Medical Microbiology, Erzurum Regional Training & Research Hospital, Erzurum, Turkey

**Keywords:** COVID-19, vitamin D, children

## Abstract

**Objective:**

The studies on children with COVID-19 are very limited. The aim of this study is to reveal the effect of serum 25-hydroxy vitamin D level on clinical and laboratory parameters.

**Materials and Methods:**

The study included 74 children (35 boys and 39 girls) diagnosed with COVID-19. The retrospective data were obtained from the file records of the patients. Seventy-four patients were divided into 3 groups (group 1, deficient; group 2, insufficient; and group 3, sufficient) according to their serum 25-hydroxy vitamin D levels.

**Results:**

The mean age of all patients was 113.25 ± 64.55 months. The mean leucocyte count was substantially higher in group 3 compared to groups 1 and 2 (*P *= .05 and *P *= .002, respectively). The mean lymphocyte and platelet count in group 3 was remarkably higher than both groups 1 and 2 (*P *= .001 and *P *= .002; and *P *= .04 and *P *= .01, respectively). The mean serum parathyroid hormone concentration in group 1 was markedly higher than both groups 2 and 3 (*P* = .003 and *P *= .002, respectively) while the mean serum 25-hydroxy vitamin D level in group 1 was remarkably lower than both groups 2 and 3 (*P* = .001 and *P* = .001, respectively). Serum 25-hydroxy vitamin D concentrations were positively correlated with leucocyte, lymphocyte, and platelet counts (*r* = 0.221, *P* = .05; *r* = 0.396, *P* = .001; and *r* = 0.249, *P* = .03, respectively) while there was a negative correlation with parathyroid hormone concentrations (*r* = −0,436, *P* = .001).

**Conclusion:**

This study suggests that COVID-19 has a benign course in children and that serum 25-hydroxy vitamin D concentration may have a role in the lymphocyte count.

**Main Points**

- This is the first study to demonstrate the relationship between serum 25-hydroxy vitamin D (25OHD) level and lymphocyte counts in children with COVID-19.
- COVID-19 appears to have a benign course in children.
- Serum 25OHD concentration may play a role in lymphocyte count in children with COVID-19.

## Introduction

Novel coronavirus disease (COVID-19) caused by “severe acute respiratory syndrome coronavirus 2 (SARS-CoV-2)” is a respiratory and systemic disorder characterized by a broad clinical spectrum ranging from mild respiratory symptoms to severe lung damage, multiple organ failure, and death.^1^ To enter the body, this virus primarily infects type II pneumocytes and enterocytes. This process is accomplished by binding the spike proteins of the virus to angiotensin-converting enzyme 2 (ACE-2) receptors on the surface of the cells.^2,3^ This receptor is expressed in many tissues throughout the body, including the lung, kidney, gastrointestinal tract, and cardiovascular, neurological, hematopoietic, and immune systems.^4,5^ The extensiveness of the ACE-2 receptors is responsible for systemic involvement in COVID-19 patients. Since lymphocytes express this receptor on their surface, they are one of the cells targeted by SARS-CoV-2. Several studies in COVID-19 adult patients have shown that lymphopenia is the most common hematological abnormality and has prognostic potential.^5^ Most immune cells, including lymphocytes, also have both CYP27B1 and vitamin D receptor expression, which means they can produce 1,25-dihydroxyvitamin D and be exposed to vitamin D, respectively.^6^ So, serum vitamin D concentration may affect immunological responses. As a matter of fact, this relationship has been revealed in many studies.^7-10^ In addition, the findings in adults with COVID-19 suggest that low serum 25-hydroxy vitamin D (25OHD) concentration is related to lymphopenia, severe clinical course, and high morbidity and mortality.^1,11-14^ The relationships between serum 25OHD level and clinical and laboratory parameters in children with COVID-19 have been investigated in only one study.^15^ That study of 29 patients has found that low 25OHD levels are only associated with fever.

In this study, we endeavored to reveal the effect of serum 25OHD level on clinical and laboratory parameters in a relatively large child population with COVID-19 by creating 3 groups (deficient, insufficient, and sufficient) according to serum 25OHD levels. The findings of our study indicate that COVID-19 has a benign course in children and that serum 25OHD concentration may have a role in the lymphocyte count, which is very important for the immune system.

## Materials and Methods

Seventy-four children (35 (47.3%) male and 39 (52.7%) female) diagnosed with COVID-19 in Eruzurum Regional Research and Training Hospital between March 2020 and September 2020 were included in the study. Real-time quantitative polymerase chain reaction (RT-qPCR) method was used in the diagnosis of COVID-19. The patients younger than 1 month and older than 18 years of age were excluded from the study. The retrospective data were obtained from the file records of the patients. Admission age; sex; comorbid disorders; clinical manifestations; examination findings; hemogram results; erythrocyte sedimentation rate; biochemical markers including magnesium, phosphorus, serum calcium, parathyroid hormone (PTH), 25OHD, alkaline phosphatase, alanine transaminase, aspartate transaminase, lactate dehydrogenase, creatinine kinase, c-reactive protein, procalcitonin, ferritin, fibrinogen, D-dimer, and troponin; radiologic findings including a posterior–anterior chest radiograph and/or a thorax tomography; and therapy modalities were evaluated. Seventy-four patients were divided into 3 groups according to their serum 25OHD levels in line with the widely accepted view.^16-19^ Group 1, group 2, and group 3 represented vitamin D inadequacy (serum 25OHD <10 ng/mL), vitamin D insufficiency (serum 25OHD <11-19 ng/mL), and vitamin D sufficiency (25OHD >20 ng/mL), respectively. Hemogram parameters were evaluated according to the ages of the patients using reference tables.^20^

### Laboratory Analyses

Full blood count was performed with the Sysmex XN-9100™ Automated Hematology System. Serum 25OHD and PTH levels were measured on the Atellica Solution (Siemens Healthineers, Erlangen, Germany) using the manufacturer`s protocol.

To diagnose SARS-CoV-2, first RNA isolation from nasopharyngeal swabs was performed using the vNAT™ solution (Bioeksen, Istanbul, Turkey) in accordance with the manufacturer’s directions. The samples were then analyzed using the Bio-speedy SARS-Cov-2 (2019-nCoV) RT-qPCR detection kit (Bioeksen) on the Bio-Rad CFX-96 (Bio-Rad Laboratories, California, USA) analyzer.

The study was conducted based on the rules of Declaration of Helsinki and approved by the Institutional Ethics Committee of Erzurum Regional Research and Training Hospital (decision no. 2020/10-115 dated May 20, 2020). An informed consent form was obtained from the patients’ parents included in the study.

### Statistical Analysis

All the calculations were made using Statistical Package for Social Sciences software version 15.0 for Windows (SPSS Inc.; Chicago, IL, USA). The Kolmogorov–Smirnov test was used for normality. The differences between groups were examined with Student’s *t*, Mann–Whitney *U*, and chi-square tests. Correlations between 2 variables were tested by Pearson’s and Spearman’s correlation coefficients. The results were expressed as the means ± standard deviation; *P* < 005 was considered statistically significant.

## Results

A total of 74 patients were included in this study. Serum 25OHD concentration was deficient (group 1) in 22 (29.7%) patients, insufficient (group 2) in 32 (43.2%) patients, and sufficient (group 3) in 20 (27.1%) patients. While 66 (89.2%) patients had a history of positive contact from their home, close relatives, and neighbors, 8 (10.8%) patients had no history of contact. The mean age of all patients was 113.25 ± 64.55 months (range: 2-210 months). The mean age in group 1 was not different from that in group 2 (*P *> .05), but it was remarkably lower in group 3 compared to both groups 1 and 2 (*P *= .001 and *P *= .001, respectively) (Table 1). When the patients were classified according to age, vitamin D deficiency increased significantly as the age increased (*P *= .001) (Table 1).

The duration of symptoms was 2.56 ± 1.76 days (range: 1-9 days). The most common clinical symptom was dry cough (45.9%), followed by fever (32.4%) and diarrhea (23%). Rale (6.8%) and rhoncus (6.8%) were the most common findings on the examination (Table 1). Three groups were not different from each other in terms of clinical symptoms, examination findings, and radiological signs (Table 1). The only case with loss of smell was a 169-month-old girl. Two girls aged 184 and 156 months had a loss of taste. Congenital heart disease, type 1 diabetes mellitus, and epilepsy were determinant comorbid disorders in 3 patients.

**Figure 1.**
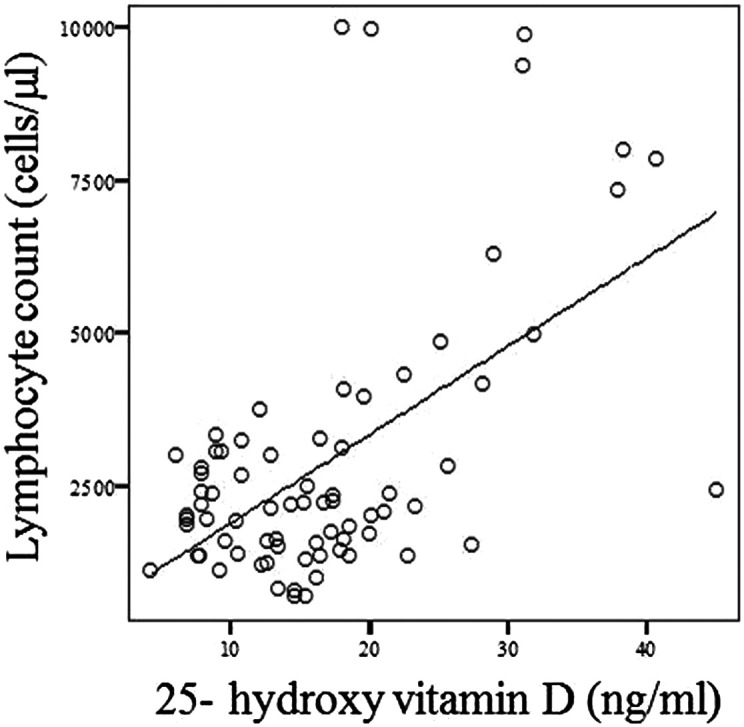
Positive correlation between serum 25-hydroxy vitamin D level and lymphocyte count (*r* = 0.369, *P *= .001).

**Figure 4.**
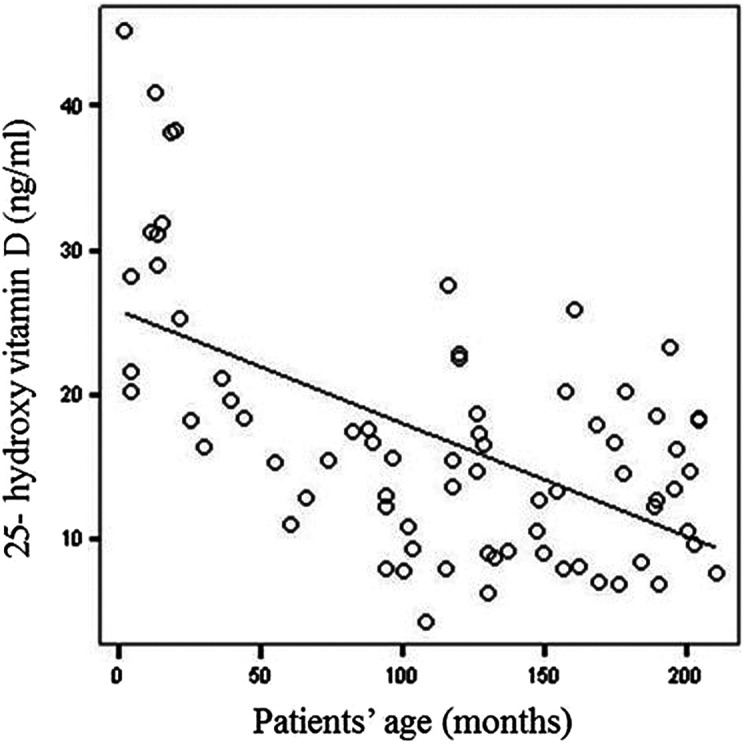
Negative correlation between patients’ age and serum 25-hydroxy vitamin D level (*r* = −0.584, *P *= .001).

Anemia, leukopenia, neutropenia, lymphopenia, and thrombocytopenia were detected in 6 (8.1%), 28 (37.8%), 30 (40.5%), 26 (35.1%), and 3 (4.0%) patients, respectively. Three groups were not different from each other in terms of prevalence of anemia, leukopenia, neutropenia, lymphopenia, and thrombocytopenia (Table 2). However, the mean leukocyte count was notably higher in group 3 compared to groups 1 and 2 (*P *= .05 and *P *= .002, respectively) (Table 3). The mean lymphocyte and platelet count in group 3 was remarkably higher than both groups 1 and 2 (*P *= .001 and *P *= .002; and *P *= .04 and *P *= .01, respectively) (Table 3). Neutrophil/lymphocyte ratio in group 3 was markedly lower than both groups 1 and 2 (*P *= .005 and *P *= .01, respectively). The mean serum PTH concentration in group 1 was appreciably higher than both groups 2 and 3 (*P *= .003 and *P *= 0.002, respectively) while the mean serum 25OHD level in group 1 was markedly lower than both groups 2 and 3 (*P *= .001 and *P *= .001, respectively). Additionally, the mean serum 25OHD concentration in group 2 was also lower than in group 3 (*P *= .001) (Table 3). Other biochemical and inflammatory findings were not different between groups. Serum 25OHD concentrations were positively correlated with leucocyte, lymphocyte, and platelet counts (*r* = 0.221, *P *= .05; *r* = 0.369, *P *= 0.001; and *r* = 0.249, *P *= 0.03, respectively) while it was negatively correlated with neutrophil/lymphocyte ratio (*r* = −0.407, *P *= .001) and PTH concentrations (*r* = −0.436, *P *= 0.001) (Figures 1-3). The patients’ age was negatively correlated with serum 25OHD level and lymphocyte and platelet count (*r* = 0.584, *P *= .001; *r* = −0.641, *P *= .001, and *r* = −0.470, *P *= .001, respectively) (Figures 4-6).

All patients had a posterior–anterior chest radiograph. Twenty-four (32.4%) patients developed pulmonary infiltrates. Computed tomography of the thorax was performed in 10 (13.5%) patients, and pneumonic consolidations were determined in 4 (5.4%) patients (Table 1).

Patients were treated with different combinations of drugs such as azithromycin, clarithromycin, amoxicillin/clavulanic acid, ampicillin/sulbactam, ceftriaxone, vancomycin, chloroquine, oseltamivir, and lopinavir/ritonavir. No patient needed intensive care support. Hospitalization time was 6.98 ± 3.93 days (range: 2-19 days). All patients were discharged in good health.

## Discussion

In this study, the idea of creating 3 categories (deficient, insufficient, and sufficient) with serum 25OHD concentration contributed to the observation of the effect of vitamin D on hemogram parameters in children with COVID-19. With this study, the relationship between serum 25OHD level and lymphocyte counts in children with COVID-19 has been revealed for the first time. The results of our study suggest that COVID-19 in children has a benign course and that the serum 25OHD concentration may have a role in the lymphocyte count, which is essential for the immune system.

Innate, adaptive, and passive immunity are essential components of the immune system. Passive immunity consists of natural and artificial immunity linked to maternal immunization and medical intervention, respectively.^21^ Physical barriers such as the respiratory or the gastrointestinal tract, skin, etc.; secretions such as mucous, gastric acid, saliva, tears, etc.; and some cells and proteins such as dendritic cells, neutrophils, macrophages, natural killer cells, mast cells, eosinophils, basophils, and complements are involved in the innate immunity while lymphocytes (T and B) are responsible for the adaptive immunity. The innate immune system is always either general or non-specific, meaning anything that is defined as foreign or non-self is a target for the innate immune response. The innate immune system is activated by the presence of antigens as well as by the chemical properties of antigens. However, the adaptive immune system is specific and activated by exposure to pathogens and uses an immunological memory to threats and accordingly enhances the immune response.^22^ The overall adaptive immune system is directed by helper T lymphocytes. B lymphocytes differentiate into plasma cells through helper T lymphocytes to initiate the humoral immune response, which is characterized by the production of a specific antibody. On the other hand, cytotoxic T lymphocytes activated by helper T lymphocytes initiate the cellular immune response and play a vital role in clearing germ-infected cells.^21^ The main players in the immune system are white blood cells, and lymphocytes are of fundamental importance because they give a specific immune response against infectious microorganisms and other foreign substances.^21,22^

Studies in COVID-19 patients show that SARS-CoV-2 primarily affects lymphocytes, resulting in lymphopenia in clinical settings.^5^ This virus invades host human cells by binding to ACE-2 receptor.^23^ Since lymphocytes express this receptor on their surface, SARS-CoV-2 can infect them directly and eventually cause them to decompose.^24^ Furthermore, the cytokine storm, characterized by outstandingly increased levels of cytokines such as interleukin (IL)-6, IL-2, IL-7, interferon-γ inducible protein 10, granulocyte colony-stimulating factor, monocyte chemoattractant protein-1, macrophage inflammatory protein-1α, and tumor necrosis factor-alpha (TNF-α), may induce lymphocyte apoptosis.^25-27^ Atrophy of lymphoid organs due to substantial cytokine activation is another contributing factor to lymphopenia because it disrupts lymphocyte turnover.^28^ The findings from adult patients with COVID-19 indicate lymphopenia is a major laboratory finding with prognostic potential.^5^

Vitamin D exerts important regulatory functions on both innate immune system and adaptive immunity because most immune cells, including macrophages, dendritic cells, and activated B and T lymphocytes, have expression of both 1-α hydroxylase and vitamin D receptors.^6^ Vitamin D interacts with the innate immune system by increasing macrophage differentiation and the production of antimicrobial peptides such as cathelicidin and human β-defensins, and reducing antigen presentation, dendritic cell maturation, and the production of cytokines such as IL6, IL12, TNF-α, NF-kB, etc. On the other hand, it has also an influence on the adaptive immune system by increasing the number of T regulator and T2 lymphocytes and the production of some cytokines such as IL4, IL5, IL10, etc., and reducing the number of T1 and T17 helper lymphocytes and the cytokines synthesis such as IL2 and interferon-γ.^29^ In that way, vitamin D may also act as an adjunct to prevent cytokine release syndrome (cytokine storm) by inhibiting the proliferation of T1 and T17 helper lymphocytes and suppressing the release of proinflammatory cytokines from both the innate and adaptive immune systems. Therefore, vitamin D is nowadays considered an immunomodulating hormone, and its serum concentration may affect immunological responses. Indeed, low vitamin D levels have been reported in the pathogenesis of many infectious diseases such as tuberculosis and microbial agents such as influenza, Human Immunodeficiency Virus, Epstein Barr Virus, Hepatitis C Virus, parasites, and fungi.^9^ The findings of previous studies suggest that decreased serum active vitamin D (calcitriol) level may contribute to reduced natural killer (NK) activity in patients with chronic diseases, and supplementation of vitamin D can remarkably increase the cytotoxicity and exocytosis of NK cells.^7,8^ On the other hand, high serum vitamin D levels are associated with higher lymphocyte count. In a study conducted by Tabatabaeizadeh et al.^10^ 580 healthy adolescent girls were given 50 000 IU capsules of cholecalciferol 9 times for 3 months at weekly intervals. After this intervention, it was found that the percentage of lymphocytes increased significantly. Similarly, most studies in adults with COVID-19 show that low serum 25OHD concentrations are associated with lymphopenia, severe clinical course, and high morbidity and mortality.^1,11-14^ There is only one study investigating the relationships between serum 25OHD level and clinical and laboratory parameters in children with COVID-19.^15^ In that study, 29 patients with COVID-19 with a lower serum 25OHD concentration were compared with 11 healthy controls. The authors found that inadequate and insufficient vitamin D levels were only associated with fever. To our knowledge, this is the first study to investigate the clinical and laboratory findings in a relatively large number of children with COVID-19 with 3 different serum 25OHD levels. This study shows that SARS-CoV-2 infection in children is related with a low lymphocyte count as in adult patients and that serum 25OHD concentration may affect lymphocyte count. The positive correlation between serum 25OHD level and lymphocyte count supports this idea. Additionally, the negative correlation between serum 25OHD and PTH levels can be considered an indirect finding indicating the true state of 25OHD in our patients. In this study, the patient’s age was negatively correlated with serum 25OHD level and lymphocyte and platelet counts. These findings suggest that as children with COVID-19 grow older, serum 25OHD decreases, and these cells decrease accordingly. However, there was no difference between groups in terms of the clinical picture and other laboratory findings. These findings suggest that lymphocyte and platelet functions are preserved and the low lymphocyte count may be an indirect effect of SARS CoV-2 resulting in manufacturing defect rather than a direct effect of SARS CoV-2 leading to cellular lysis. The fact that this virus invades lymphocytes by binding to the ACE-2 receptor and that this receptor is less mature in young people may support our view.^23-30^ Accordingly, the clinical severity and mortality rate of the disease in children may be lower than in adults. In our study, no deaths occurred and all patients were discharged in a healthy state.

This study has a couple of limitations. First, some patients had some missing laboratory data due to a retrospective study design. Second, some clinical findings such as loss of smell and taste could not be assessed in young children. However, our study may shed light on future studies on the relationship between vitamin D deficiency and COVID-19.

In conclusion, this study is the first to demonstrate the relationship between serum 25OHD level and lymphocyte counts in children with COVID-19. The results of this study suggest that COVID-19 has a benign course in children and that serum 25OHD concentration may have a role in the lymphocyte count, which is very important for the immune system.

## Figures and Tables

**Figure 2. f2-eajm-54-3-285:**
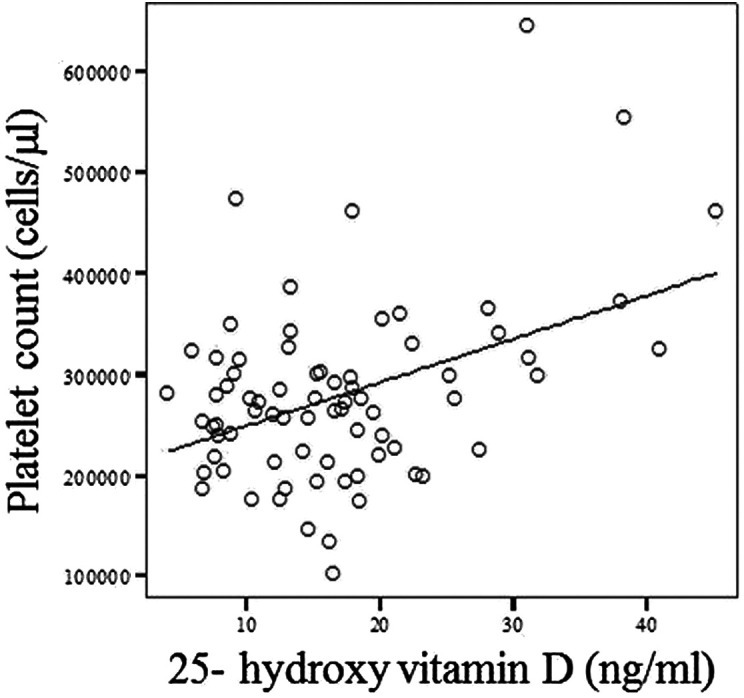
Positive correlation between serum 25-hydroxy vitamin D level and platelet count (*r* = 0.249, *P *= .03).

**Figure 3.**
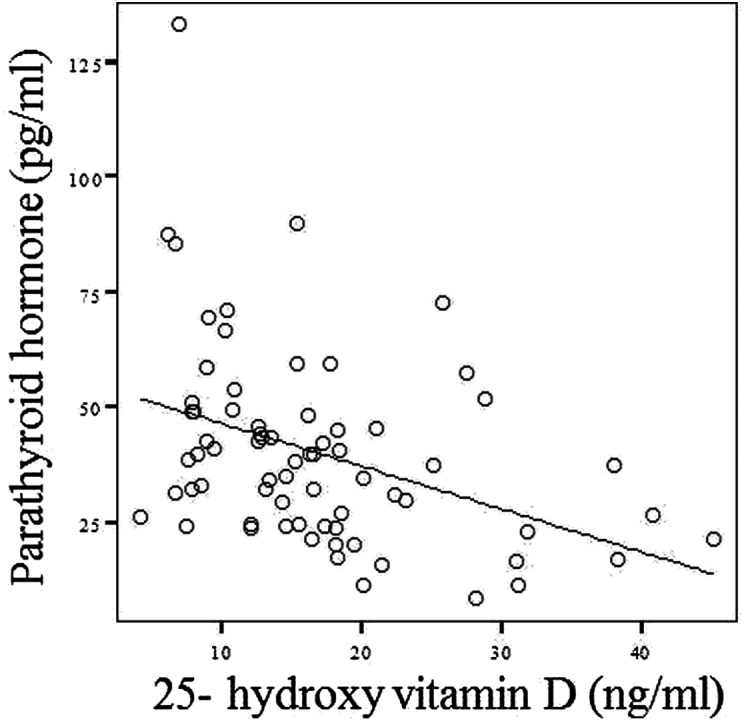
Negative correlation between serum 25-hydroxy vitamin D and parathyroid hormone levels (*r* = −0.436, *P *= .001).

**Figure 5. f5-eajm-54-3-285:**
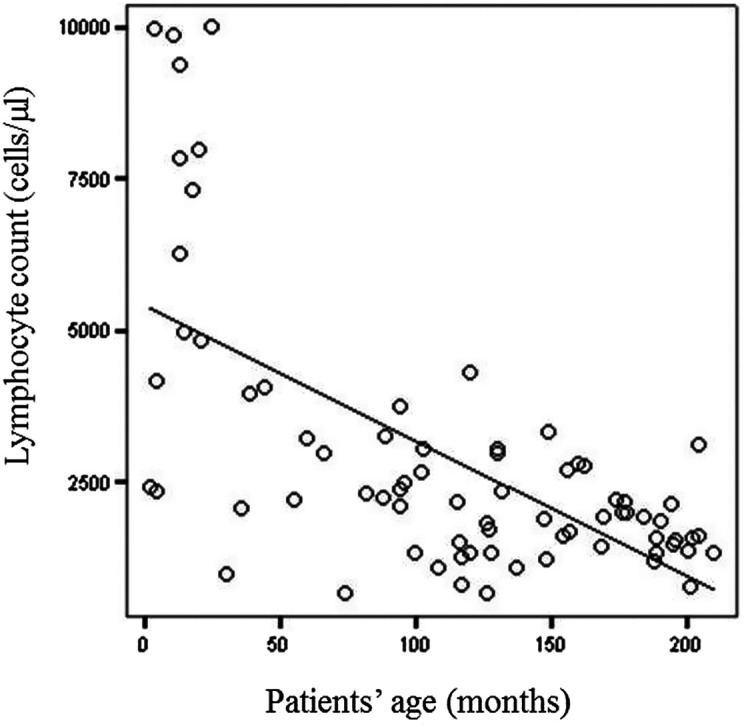
Negative correlation between patients’ age and lymphocyte count (*r* = −0.641, *P *= .001).

**Figure 6.**
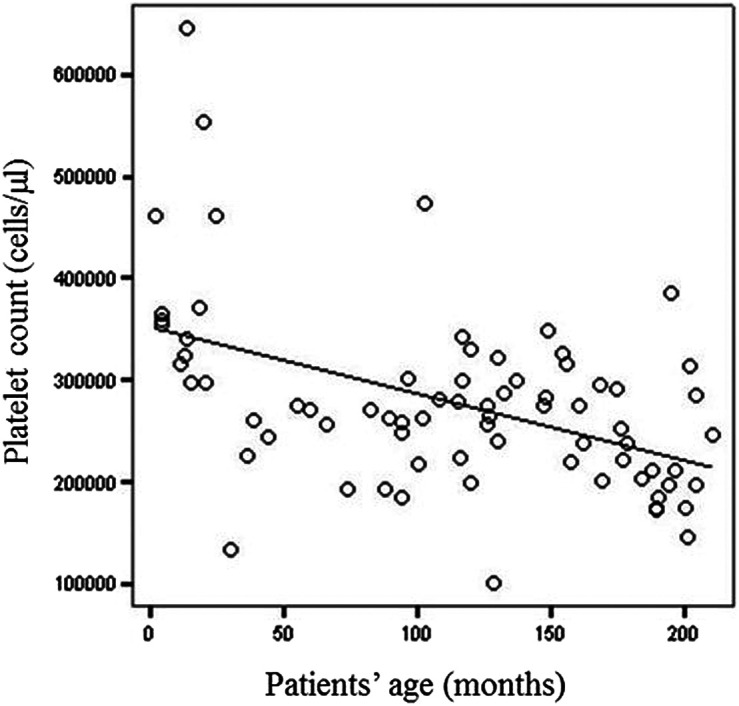
Negative correlation between patients’ age and platelet count (*r* = −0.470, *P *= .001).

**Table 1.** The Clinic and Laboratory Characteristics of Children with COVID-19

G1-G2	G1-G3	G2-G3
Characteristic	All Patients (n = 74)	Group 1 (G1) (n = 22)	Group 2 (G2) (n = 32)	Group 3 (G3) (n = 20)	P*		
					**The mean age (months) ± SD**	113.25 ± 64.55	143.45 ± 40.45	125.12 ± 56.81	61.05 ± 69.08	.199	.001	.001
**Age groups**					***P*** **		
0-72 months, n (%)	22 (29.7)	1 (4.1)	8 (25)	13 (65)	**.001**		
73-144 months, n (%)	28 (37.8)	12 (54.5)	13 (40.6)	3 (15)			
145-216 months, n (%)	24 (32.4)	9 (40.9)	11 (34.3)	4 (20)			
**Clinical symptoms**							
Dry cough, n (%)	34 (45.9)	8 (36.3)	15 (46.8)	11 (55)	.47		
Fever >38°C, n (%)	24 (32.4)	6 (27.2)	9 (28.1)	9 (45)	.37		
Diarrhea, n (%)	17 (23)	2 (9.0)	8 (25)	7 (35)	.12		
Headache, n (%)	10 (13.5)	1 (4.5)	7 (21.8)	2 (10)	.16		
Sore throat, n (%)	10 (13.5)	4 (18.1)	5 (15.6)	1 (5)	.41		
Dyspnea, n (%)	9 (12.2)	1 (4.5)	7 (21.8)	1 (5)	.08		
Myalgia, n (%)	9 (12.2)	1 (4.5)	7 (21.8)	1 (5)	.08		
Vomiting, n (%)	5 (6.8)	2 (9.0)	3 (9.3)	0 (0)	.37		
Runny nose, n (%)	4 (5.4)	2 (9.0)	2 (6.2)	0 (0)	.41		
Loss of taste, n (%)	2 (2.7)	2 (9.0)	0 (0)	0 (0)	.08		
Loss of smell, n (%)	1 (1.4)	1 (4.5)	0 (0)	0 (0)	.30		
**Examination findings**							
Rale, n (%)	5 (6.8)	1 (4.5)	4 (12.5)	0 (0)	.19		
Rhoncus, n (%)	5 (6.8)	0 (0)	4 (12.5)	1 (5)	.18		
Tachypnea, n (%)	3 (4.1)	0 (0)	2 (6.2)	1 (5)	.50		
Retractions on chest	2 (2.7)	0 (0)	1 (3.1)	1 (5)	.59		
Tachycardia, n (%)	1 (1.4)	1 (0)	0 (0)	0 (0)	.51		
**Radiological signs**							
PA chest x‐ray, n (%)	24 (32.4)	6 (27.2)	13 (40.6)	5 (25)	.41		
Chest CT n (%)	4 (5.4)	1 (4.54)	3 (9.37)	0 (0)	.40		

PA, posterior–anterior; CT, computerized tomography.

*Student’s *t*-test was used.

**Chi-square test was used.

**Table 2.** The Prevalence of Cytopenia in Children with COVID-19

	Group 1 (n = 22)	Group 2 (n = 32)	Group 3 (n = 20)	*P*
Anemia, n (%)	2 (9)	1 (3.1)	3 (15)	.32
Leukopenia, n (%)	7 (31.8)	14 (43.7)	7 (35)	.64
Neutropenia, n (%)	8 (36.3)	12 (37.5)	10 (50)	.59
Lymphopenia, n (%)	6 (27.2)	14 (43.7)	6 (30)	.39
Thrombocytopenia, n (%)	0 (0)	3 (9.3)	0 (0)	.12

**Table 3.** Laboratory Findings of Children with COVID-19

G1-G2	G2-G3	G1-G3
Findings	All Patients	Group 1 (G1)	Group 2 (G2)	Group 3 (G3)	*P*		
					**Complete blood count**							
Hb (g/dL), n = 74	13.43 ± 1.61	13.63 ± 1.37	13.84 ± 1.40	13.26 ± 1.89	.45	.52	.34
Leucocyte (cells/μL), n = 74	6414.32 ± 3274.61	6024.09 ± 2555.99	5524.68 ± 2304.53	8267.0 ± 4493.45	.45	**.002**	**.05**
Neutrophil (cells/μL), n = 74	2810.27 ± 1897.29	3218.18 ± 2175.95	2666.25 ± 1570.26	2592.0 ± 2072.81	.28	.88	.34
Lymphocyte (cells/μL), n = 74	2887.70 ± 2250.52	2191.36 ± 708.65	2192.18 ± 1703.15	4766.50 ± 3013.30	.99	**.002**	**.001**
Neutrophil/lymphocyte	1.48 ± 1.47	1.84 ± 1.81	1.64 ± 1.22	0.73 ± 0.70	.64	**.01**	**.005**
PLT (cells/μL), n = 74	277 824.32 ± 89 701.99	269 772.72 ± 64 503.17	251 000.00 ± 73 393.32	329 600.0 ± 115 841.72	.33	**.01**	**.04**
**Inflammatory**							
CRP, mg/dL, n = 68	8.02 ± 21.42	6.98 ± 18.01	10.38 ± 28.46	5.26 ± 6.67	.63	.35	.70
Procalcitonin, n = 46	0.10 ± 0.21	0.09 ± 0.17	0.11 ± 0.26	0.06 ± 0.05	.73	.33	.63
ESR (mm/h), n = 44	7.27 ± 5.94	8.5 ± 5.83	7.20 ± 6.93	5 ± 2.07	.55	.21	.11
Ferritin, n = 47	48.04 ± 34.01	49.08 ± 45.15	48.64 ± 28.32	44.10 ± 18.29	.97	.67	.76
D-dimer, n = 52	0.61 ± 0.82	0.66 ± 0.99	0.66 ± 0.81	0.41 ± 0.45	.97	.27	.45
Fibrinogen, n = 37	277.64 ± 85.07	283.46 ± 45.95	295.06 ± 102.96	233.37 ± 88.8	.71	.16	.10
**Biochemical**							
AST (U/L), n = 73	30.21 ± 19.26	26.77 ± 20.12	26.93 ± 16.72	29.73 ± 20.03	.69	.95	.74
ALT (U/L), n = 73	21.20 ± 17.65	21.22 ± 12.97	18.90 ± 20.58	25.05 ± 17.18	.64	.25	.42
LDH (U/L), n = 71	288.88 ± 179.11	305.27 ± 294.8	272.12 ± 86.98	299.23 ± 102.89	.55	.33	.93
CK, n = 67	111.52 ± 73.19	102.35 ± 63.11	107.65 ± 68.31	131.12 ± 93.4	.77	.33	.26
Troponin, n = 57	0.26 ± 0.76	0.06 ± 0.22	0.36 ± 0.92	0.27 ± 0.78	.21	.78	.32
Mg (mg/dL), n = 72	1.69 ± 0.22	1.94 ± 0.18	1.93 ± 0.22	2.03 ± 0.26	.83	.14	.18
Zn (mg/dL), n = 62	120.79 ± 44.85	128.31 ± 46.85	115.44 ± 32.26	121.64 ± 63.33	.26	.73	.73
Ca (mg/dL), n = 73	9.68 ± 0.46	9.6 ± 0.43	9.69 ± 0.45	9.77 ± 0.5	.51	.53	.25
P (mg/dL), n = 73	4.66 ± 0.74	4.62 ± 0.64	4.55 ± 0.83	4.9 ± 0.67	.71	.10	.18
ALP (U/L), n = 73	237.04 ± 107.24	210.09 ± 91.19	228.37 ± 101.63	282.84 ± 123.59	.50	.11	.03
PTH (pg/mL), n = 70	40.07 ± 21.20	53.76 ± 25.48	36.47 ± 14.93	30.29 ± 17.64	**.003**	.22	**.002**
25OHD (ng/dL), n = 74	16.90 ± 8.67	8.34 ± 1.63	15.72 ± 2.21	28.21 ± 7.51	**.001**	**.001**	**.001**

PLT, platelet; CRP, c-reactive protein; ESR, erythrocyte sedimentation rate; AST, aspartate transaminase; ALT, alanine transaminase; LDH, lactate dehydrogenase; CK, creatinine kinase; Mg, magnesium; Zn, Zinc; Ca, calcium; P, phosphorus; ALP, alkaline phosphatase; PTH, parathyroid hormone; 25OHD, 25-hydroxy vitamin D.
